# The bidirectional relationship between endometriosis and microbiome

**DOI:** 10.3389/fendo.2023.1110824

**Published:** 2023-03-07

**Authors:** Cansu Uzuner, Jason Mak, Fatima El-Assaad, George Condous

**Affiliations:** ^1^ Endometriosis Ultrasound and Advanced Endosurgery Unit, Sydney Medical School Nepean, University of Sydney, Nepean Hospital, Sydney, NSW, Australia; ^2^ University of New South Wales Microbiome Research Centre, St. George and Sutherland Clinical Campuses, School of Clinical Medicine, Faculty of Medicine and Health, University of New South Wales, Sydney, NSW, Australia

**Keywords:** endometriosis, microbiome, microbiota, dysbiosis, inflammation, immune response

## Abstract

Endometriosis has been described by many different theories of pathogenesis over the years. It is now also appreciated to be a state of chronic inflammation, and the role of immune dysfunction in its development has been proven. There is increasing evidence to support the role of the microbiome in the formation and progression of endometriosis *via* inflammatory pathways. The dysbiosis seen in endometriosis is thought to be both causative and a consequence of the pathogenesis. Gut, peritoneal fluid and female reproductive tract microbiota has been studied to understand if there are any microbiome signatures specific to endometriosis. New research on how to manipulate the microbiome for better detection and treatment of endometriosis is emerging.

## Introduction

Endometriosis is an inflammatory disease characterized by the presence of endometrium-like tissue outside of the endometrium and myometrium (1, 2). It has a variety of subtypes and clinical presentations, ranging from being asymptomatic to causing chronic pain and infertility (2, 3). Endometriosis affects a significant proportion of the world’s population – estimated to be present in up to 10% of females, and up to 50% of women with infertility (1). It also has significant healthcare costs, with the most recent study in 2022 appraising the direct cost of endometriosis to be US$1459 to US$20,239 per patient per year, and indirect cost to be between US$4,572 and US$14,079 (4). An Australian study in 2019 projected the total economic burden per year in the reproductive aged population (at 10% prevalence) to be Int$6.50 billion (5).

Recently, an increased understanding of the role of microbiota and immune dysbiosis in many diseases has also brought to light the possibility of their role in development of endometriosis. The knowledge on the human microbiome and its definition has been rapidly expanding due to new developments in sequencing methods and analytical techniques (6). With this growing field, terminology can lead to confusion. Microbiota is defined as the community of microorganisms living in or on the human body site – it includes bacteria, archaea (single celled organisms without nuclei), fungi, eukaryotes and viruses. Microbiome is defined as the collective genomes of these microbes (7, 8). Microbiota has an associated theatre of activity – structural elements, metabolites, signal molecules, and the surrounding environmental conditions – that supports local immune, metabolic and epithelial function (9). When there is dysbiosis – defined as an imbalance or impairment of the microbiota – this support breaks down (3, 9, 10). Microbes and their metabolites can translocate to different body sites and can trigger an immune response and inflammation that is involved in multitude of diseases such as metabolic disorders, many neurological disorders, arthritis, psoriasis, inflammatory bowel disease and cancer (10–12).

## Pathogenesis of endometriosis

There have been many postulations of pathogenesis of endometriosis including retrograde menstruation, immune dysfunction, inflammation, hormone dysregulation, coelomic metaplasia, lymphatic or hematological metastasis, stem cell dysfunction and genetic and epigenetic factors (13–16). A combination of these theories is likely in play together to lead to this chronic disease.

### Immunopathology of endometriosis

Peritoneal endometriosis has been described as a chronic inflammatory state (17, 18). It is thought that the inflammatory state and the immune dysfunction in the peritoneum are both the cause and result of endometriosis. Immune dysregulation also leads to poor immunosurveillance as an appropriate response cannot be mounted to the refluxed endometrial cells and debris. This allows ectopic endometrial cells to persist in the peritoneal cavity (10, 19).

Firstly, the peritoneal inflammation plays a role in the development of the disease as well as the symptomology of the disease – pain and subfertility (17). The theory of retrograde menstruation is the most widely accepted pathogenesis of endometriosis (15). However, this theory alone does not explain disease prevalence, as women without endometriosis also display retrograde menstruation. Once endometrial cells are in the peritoneal cavity, they are required to adhere and proliferate to lead to endometriosis (10, 19). The inflammation and altered immunity create the right environment for cellular adhesion and endometriosis development and disease progression (10, 17, 18).

Oxidative stress is thought to be a key contributor to this inflammatory process (18, 20, 21). Reactive oxygen species (ROS) are intermediaries produced by the normal oxygen metabolism and have been implicated in his process (17, 18, 21). As a protective mechanism, cells cultivate antioxidant systems to counteract the ROS. When there is an imbalance between the ROS and antioxidants, with an abundance of ROS and deficiency in antioxidants, oxidative stress occurs (18, 20). In endometriosis, this imbalance is postulated to arise from erythrocytes in the peritoneal cavity and their toxic by-products of heme and iron. Free heme and iron lead to formation of ROS (17, 18, 21). This oxidative stress not only leads to cellular damage but also can alter cellular function *via* affecting protein activity and gene expression. The transcription factor called nuclear factor kappa-B (NF-κB) induces expression of multiple genes encoding proinflammatory cytokines, growth factors, adhesion molecules and enzymes, and has been implicated in peritoneal endometriosis by aiding in endometrial cell adhesion, proliferation and neovascularization (10, 17, 21–23).

Once the endometriotic implants adhere to the peritoneum, they require the help of cytokines and growth factors such as vascular endothelial growth factor (VEGF), tumour necrosis factor-α (TNF-α) and interleukin-8 (IL-8) for angiogenesis and lesion proliferation (10, 19, 24). These are primarily expressed by macrophages in the peritoneal cavity *via* increased activation of NF-κB pathways. There have been studies that support this theory by demonstrating increased number of macrophages, monocytes, and inflammatory mediators such as complements and cytokines in the peritoneal fluid of women with endometriosis (25, 26).

The dysregulation of both the innate and adaptive immunity are involved in the immunopathology of endometriosis. It has been shown that ectopic endometrial deposits, compared to matched eutopic endometrium of the same patients as well as to the endometrial tissues of the control group, have elevated expression of molecular genes associated with immune system process activation (27). Genes encoding for proinflammatory cytokines and receptors, cell adhesion molecules, complement proteins and angiogenesis are increased, and genes involved in regulation of inflammation, NK and cytotoxic T-cell activity, and cellular apoptosis are aberrantly expressed in ectopic endometrial tissues (27). These findings support the immune dysregulation in endometriosis.

### Role of genetics and epigenetics in pathogenesis of endometriosis

Higher rates of endometriosis are seen in the relatives of women affected with endometriosis (28, 29). Twin studies have also supported the genetic influences on endometriosis by demonstrating a concordance ratio of 2:1 between monozygotic and dizygotic twins and a genetic risk ratio of 2.34 for endometriosis for a sibling, as well as 47-51% of endometriosis variation to be attributable to additive genetic effects (30, 31). As our understanding of genes and their role in disease has exponentially grown, there have been many studies conducted to determine the genes involved in specific condition such as endometriosis. Genome-wide association studies (GWAS) have discovered up to 27 significant loci associated with endometriosis but the challenge of understanding the functional consequences of these loci remain (32–35). There are genes associated with steroidogenesis and sex hormone receptorial activity, leading to dysregulation of estrogen and progesterone receptor ligand signaling, genes involved in inflammation and immune response, neoangiogenesis and DNA reparation, and genes coding for metabolism regulation and cell growth postulated to be instrumental in establishment of endometriosis (33). Furthermore, genes have been shown to regulate their neighboring genes by epigenetic mechanisms. Epigenetics is defined as heritable changes in gene function that are not associated with DNA sequence changes but involves processes such as DNA methylation and histone modification (34, 36). Epigenetic mechanisms have been demonstrated to be involved in regulating immune processes such as cytokine expression, T-cell differentiation, antigen presentation and regulation of transcription factors, such as NF-κB, which has been implicated in immunopathogenesis of endometriosis (10, 36, 37). The genetic and epigenetic theory is also supported by the finding that there are gene expression and molecular differences found in the endometrium of women with endometriosis is compared to the endometrium of healthy controls, as well as between the eutopic and ectopic endometrium of women with endometriosis (36, 38–40).

## Microbiome of endometriosis

Alterations in the microbiota of gut, peritoneal fluid and female reproductive tract in subjects with endometriosis compared to healthy controls have been demonstrated in increasing number of both human and animal studies (10, 41–51). It is not too clear whether these alterations are a result of endometriosis or whether they are the cause of endometriosis. However experimental animal models support a bidirectional relationship between endometriosis and microbiota changes (42, 50). In a particular study in mice who had surgically induced endometriosis, a reduction in the size of the endometriotic lesions was seen after treatment with antibiotics. After fecal microbiota transfer from endometriotic mice, regrowth of the lesions and associated inflammation was seen (42).

### Gut microbiota

Gut microbiota has been the most studied body site in endometriosis microbiome research. The gut microbiome is dominated by bacteria, especially the members of the phyla *Bacteroidetes* and *Firmicutes*. In most healthy humans, percentage of each of these two dominant phyla can vary but the combined percentage tends to be approximately 95%. In a disease state, the gut microbiome can shift to represent large percentages of other bacterial phyla, such as *Proteobacteria*, *Verrucomicrobia*, *Actinobacteria*, or *Fusobacteria* (7, 52).

Interestingly, a systematic review conducted in 2019 on the microbial signatures of endometriosis found the following results (3). At the phylum level, *Actinobacteria*, *Firmicutes*, *Proteobacteria* and *Verrucomicrobia* were identified as being significantly higher in the gut of the endometriosis cohort, compared with controls. In contrast, *Lactobacillaceae* was found to be significantly decreased. They concluded that the levels of *Proteobacteria*, *Enterobacteriaceae*, *Streptococcus* and *E. coli* were elevated across various microbiome sites in endometriosis cohorts.

Since this systematic review, a number of studies exploring the role of gut microbiome in endometriosis have been published (41, 48, 53). Svensson et al’s 2021 study did not find any significant differences in the abundance of bacterial classes between patient with or without isolated ovarian endometriosis, involvement of the gastrointestinal tract, gastrointestinal symptoms, or hormonal treatment (48). However, other studies have demonstrated that in the gut microbiota, more women in the endometriosis group had *Shigella and Escherichia* dominance (41).

### Female reproductive tract microbiota

The female reproductive tract microbiota can be divided into the vagina, cervix, endometrium, fallopian tubes and ovaries. Majority of the studies in the microbiota of the female reproductive tract in endometriosis have focused on the cervix and the vagina. It has been found that the distribution of microbiota is similar in the cervical mucus of women with and without endometriosis regardless of the phases of the menstrual cycle, however the abundance of each changes (54). *Lactobacilli* is the predominant species in the vagina and the cervix. In addition to this, the abundance of *Corynebacterium*, *Enterobacteriaceae*, *Flavobacterium*, *Pseudomonas*, and *Streptococcus* are increased in the endometriosis group compared to the control group, with *Enterobacteriaceae* and *Streptococcus* being the more noteworthy candidates (54). A recent review has summarized that bacterial vaginosis-associated bacteria and Lactobacillus depletion in the cervicovaginal microbiome were associated with endometriosis and infertility in the majority of studies they analyzed (53). Another noteworthy finding is that reduced richness and diversity of cervical microbiome were detected in patients with more severe endometriosis symptoms including higher CA125 levels, more severe pain and infertility (55). This study suggested that cervical microbiome has an important role in regulating the pathogenesis of the associated complications of endometriosis and concluded that a more diverse cervical microbiome is associated with better clinical outcomes.

A small study of 14 participants with Stage III-IV endometriosis and 14 healthy controls revealed that the vaginal, cervical and gut microbiota composition among the endometriosis group were similar; but it showed that some potentially pathogenic species were increased in the cervical and stool microbiome in women with endometriosis compared to the control group (41). In the cervical microbiota, *Gardnerella*, *Streptococcus*, *Escherichia*, *Shigella*, and *Ureoplasma* were increased. Interestingly they observed a total absence of a particular genus, *Atopobium* in vaginal and cervical microbiota. *Atopobium* has been recently implicated as a gynecological pathogen potentially associated with endometrial cancer, and lower incidence of it was seen in women with benign gynecological pathologies (56). It is unclear that this association is causal or coincidental. It could be proposed that the absence of *Atopobium* can be related to occurrence benign gynecological pathologies, in which endometriosis a part of.

Less studies have been performed exploring the relationship with viruses, particularly human papilloma virus (HPV) with endometriosis (3). Majority of these studies have found HPV detection to be higher and therefore associated with endometriosis (57–59).

### Peritoneal microbiota

Microbiota diversity of the peritoneal fluid was shown to be similar in women with an without endometriosis (51). However, the abundance of these microbiota differed (47, 51). *Acidovorax*, *Devosia*, *Methylobacterium*, *Phascolarctobacterium*, and *Streptococcus* were more abundant in the peritoneal fluid of endometriosis patients than the controls, while *Brevundimonas* and *Stenotrophomonas* were less abundant (51). Another study reported the abundance of *Acinetobacter*, *Pseudomonas*, *Streptococcus*, and *Enhydrobacter* to be significantly increased while the abundance of *Propionibacterium*, *Actinomyces*, and *Rothia* to be significantly decreased in the endometriosis group compared with those in the control group (47). A third study concluded *Sphingobium*, *Pseudomonadaceae*, *Sphingomonas*, *Acinetobacter*, *Erysipelothrix*, *Clostridiales*, *Micrococcaceae*, *Vagococcus*, *Dysgonomonas*, P*seudomonas viridiflava*, *Shewanella*, *Tissierellaceae* were enriched in the peritoneal fluid of endometriosis patients compared to the control group (49). When the microbiota of deep endometriosis lesions were examined by Hernandes et al. in 2020, *Alishewanella, Enterococcus* and *Pseudomonas* were demonstrated to be more abundant (43). *Acinetobacter* and *Pseudomonas* prove to be persistently present in several of these studies (43, 47, 49). These findings not only support that microbiome composition is altered in the peritoneal environment in women with endometriosis but also point to the possibility of finding a peritoneal fluid microbial signature specific to endometriosis.

## Microbiome’s role in pathogenesis of endometriosis

The effects of dysbiosis could be contributing to the pathogenesis of endometriosis *via* inflammation and immune modulation and there is new evidence suggesting a role of the microbiome in development of endometriosis (10, 41, 43, 49, 53, 60–62).

### Bacterial contamination theory

A “bacterial contamination” theory for endometriosis progression of endometriosis has been postulated. Khan et al. (63) examined *Escherichia coli* (*E. coli*) concentrations in the menstrual blood of women with endometriosis in comparison to control groups. They found that there was increased number of *E. coli* colony formation in women with endometriosis, especially those with peritoneal endometriosis in addition to ovarian endometriomas. They suggested that *E. coli* contamination of the menstrual blood would be a constant source of bacterial endotoxin or a lipopolysaccharide in the peritoneal cavity. The primary inflammation caused by the lipopolysaccharides would lead to the secretion of secondary inflammatory mediators such as NF-κB in the peritoneal cavity *via* promoting Toll-like receptor 4 (TLR4) which are present on macrophages and other immune cells (60). This would start the cascade of endometriosis development as explained with the immunopathology of endometriosis.

The bacterial contamination theory has since been discussed (10, 60) and is supported by other research demonstrating increased levels of *Proteobacteria*, which is a phylum of bacteria that produces lipopolysaccharides, in endometriosis cohorts (41, 45, 50, 54, 63, 64). Furthermore, a large cohort study of over 140,000 women demonstrated that there is a three-fold increased risk of developing endometriosis in women with a history of pelvic inflammatory disease (PID) compared to the control cohort (62). A similar result has been exhibited by another study in which double the incidence of endometriosis was seen in women with a lower genital tract infection (61).

### Estrobolomes in development of endometriosis

Another possible mechanism of how microbiome can influence endometriosis development and progression can be explained by the altered estrogen metabolism that is seen with dysbiosis (65). It is known that endometriosis is an estrogen driven condition (66, 67). Certain dysbiotic gut microbiota are known as ‘estrobolomes’ whose products can metabolize estrogen, increasing the circulating levels of estrogen in the body (3, 53, 68). These estrobolomes are known to secrete β-glucuronidase and β-glucosidases which deconjugate estrogen, which in turn increases the re-absorption of free estrogens in the gut (68, 69). It is theorized that this can lead to a hyperestrogenic state and contribute to progressing of endometriosis (53, 65). Multiple genera in the gut microbiome encode for β-glucuronidase, including *Bacteroides*, *Bifidobacterium*, *Escherichia* and *Lactobacillus* (3, 69). Interestingly, some studies have found higher levels of *Bifidobacterium* and *Escherichia* in endometriosis groups over control groups (50, 63). It is also known that there is dysbiosis in the Bacteroidetes/Firmicutes ratios, which are the two dominant phyla in the gut, in women with endometriosis (10, 50).

### Genetic and epigenetic factors

Bacterial induced epigenetic deregulation of host cells has been well studied (70–72). Dysbiosis and female genital tract infections may induce genetic and epigenetic incidents, leading to increased oxidative stress and changes in the immune responses, which in turn could play a role in the formation of endometriosis (72). A study on *Mycoplasma genitalium* revealed that the gene expression of peritoneal fluid cells of women with endometriosis who are colonized with *Mycoplasma genitalium* were significantly downregulated which in turn would inhibit immune cells recruited to the site (73). Viruses are also known to be mutagenic and this has been proven in many malignancies caused by carcinogenic viruses such as HPV, human immunodeficiency virus (HIV), hepatitis B virus (HBV), hepatitis C virus (HCV) and Ebstein-Barr virus (EBV) (74–76). There have only been a few studies assessing the relationship between viruses, particularly HPV, and endometriosis, and future studies are needed in this area (57–59). Furthermore, it has been reported that inflammation itself can cause aberrant DNA methylation patterns leading to hypermethylation which in turn effects the production of certain transcription factors and receptors (such as HOXA10 and progesterone receptor B) that is seen in endometriosis (39, 77, 78).

## Clinical implications

### Performance of different body sites

Only a few studies have compared microbiota of different body sites to assess which site is the best performer in predicting endometriosis. One of the studies examined the gut, peritoneal fluid and cervical mucus. It demonstrated that the gut and peritoneal fluid have higher richness and diversity in microbiota compared to cervical mucus and concluded that the gut microbiota is the top performing predictor of endometriosis out of the three (44). Another study compared samples from lower third of vagina, posterior vaginal fornix, cervical mucus, endometrium and peritoneal fluid (49). Each site had a different microbiota distribution. Significant difference of the community diversity began showing in the cervical mucus of endometriosis patients and gradually increased upward the reproductive tract, suggesting the upper female reproductive tract is better indicator for the risk of endometriosis if used as a screening tool (49).

### Microbiome in predicting endometriosis stage

There is limited evidence available in the differences between microbiotas of different endometriosis stages and all the available evidence is based on studies with small numbers (44, 79). The studies that investigated this question used revised-ASRM (rASRM) stages (80). A study of 21 participants with endometriosis found no difference between gut microbiota of early (Stage I and II) and advanced stages (Stage III and IV) of endometriosis (44). A larger study with 59 participants (34 with endometriosis, 24 control) assessed gut and vaginal samples collected at two different time periods within the menstrual cycle (79). They reported that 9/35 (25.7%) had Stage I, 12/35 (34.2%) had Stage II, 3/35 (11.4%) had Stage III, and 10/35 (28.5%) had Stage IV endometriosis. They grouped Stage I and II together and Stage II and IV together for comparative analysis. The analysis did not show any significant differences in the microbiota between either two groups of stages of endometriosis or control groups. However, they concluded that vaginal microbiome was predictive of the stage of the disease based on an operational taxonomical unit (OTU) from the genus *Anaerococcus*. Both of these studies used 16S rRNA gene sequencing for analysis. Other studies that performed a sub-analysis on rASRM stages any briefly mentioned that there was no significant difference between the different stages (45, 73).

### Evidence so far in altering microbiome to treat endometriosis

**Figure 1:**
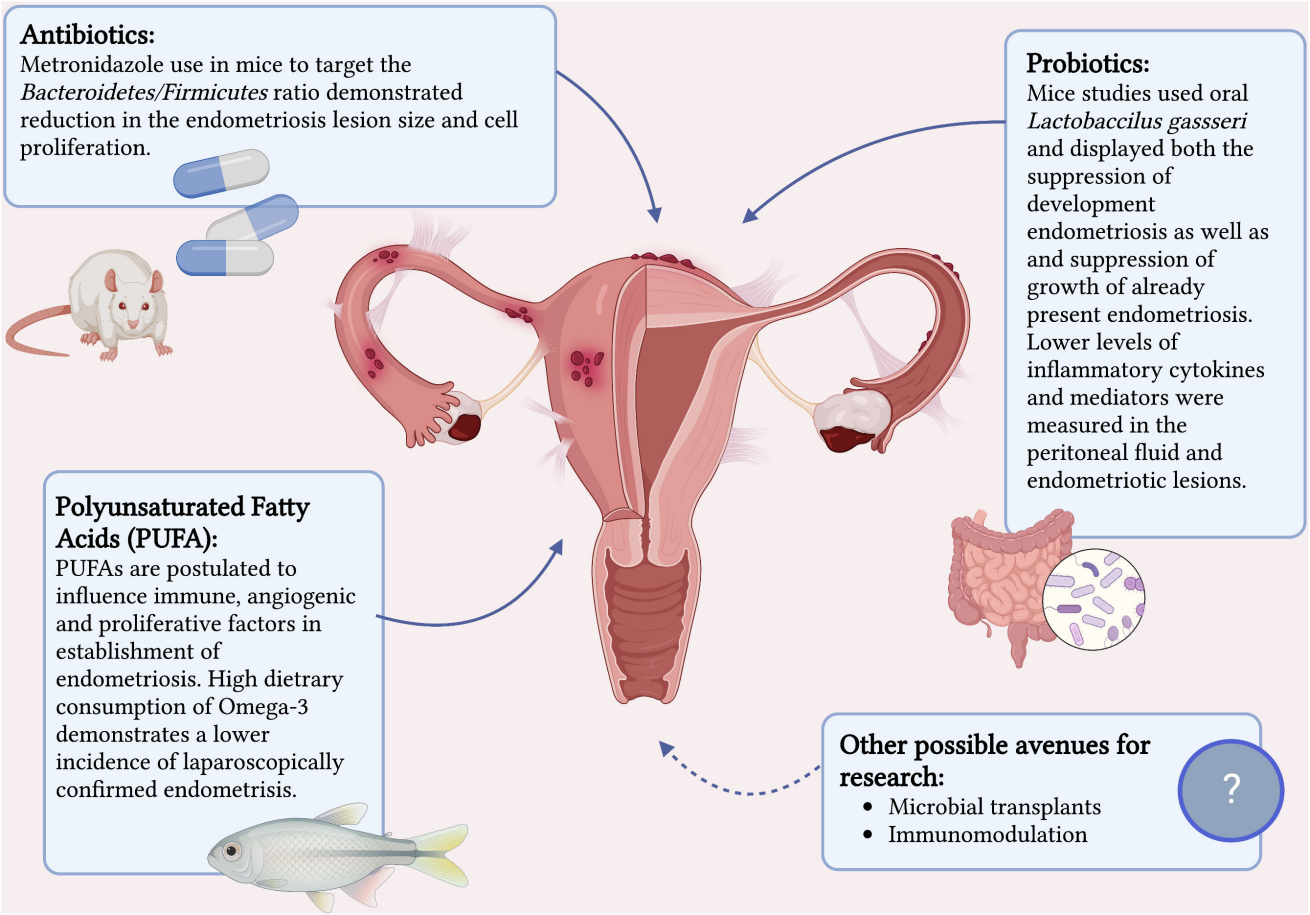
Current interventions that have a potential in treatment of endometriosis. Created with BioRender.com.

Evidence on the role of microbiome and dysbiosis in the development of endometriosis is rapidly mounting. Therapeutic manipulation of the microbiome in treatment and prevention of endometriosis is a very real possibility. Animal studies have already established this possibility (42). **Figure 1** summaries the current interventions that have a potential in treatment of endometriosis. Chadchan et al’s study on mice (42) with surgically induced endometriosis found higher levels of *Bacteroidetes* and lower levels of *Firmicutes* in the gut microbiota composition compared to the control mice. Metronidazole was used on the mice with endometriosis to target *Bacteroidetes* and this demonstrated reduction in the endometriosis lesion size and cell proliferation. Furthermore, they proved a reduced inflammatory response in the treated mice by measuring lower levels of inflammatory cytokines and mediators in the peritoneal fluid and endometriotic lesions.

Probiotics is another promising treatment option that has proven itself with other benign gynecological conditions such as candida vulvovaginitis and bacterial vaginosis (81). There have been two mice studies that show some potential in its benefit in the treatment of endometriosis (82, 83). Both studies investigated the role of oral *Lactobaccilus gassseri* and displayed both the suppression of development endometriosis as well as and suppression of growth of already present endometriosis. The mechanism postulated was immunostimulatory activity *via* activation of NK cells and reduction in development of ectopic endometriotic lesions.

Although there are limited animal studies on antibiotics and probiotics on treatment of endometriosis (42, 82, 83), there have not been any studies to date to investigate the specific role of probiotics or prebiotics in helping resolve the dysbiosis associated with endometriosis in hopes to assist in its treatment. Urgent future research is needed to study the role of probiotic and antibiotic therapy further in human subjects. Conversely it is important to remember that excessive use of antibiotics can have the adverse effect of altering healthy commensal microbiota and contributing to antimicrobial resistance.

Lastly, the therapeutic anti-inflammatory effect of polyunsaturated fatty acids (PUFAs) such as omega-3 and omega-6 on multitude of diseases is well established (84, 85) and increasing evidence on beneficial effects of PUFAs on endometriosis is becoming available. Women with endogenously higher serum PUFAs levels have been shown to be 82% less likely to have endometriosis compared to women with low PUFAs levels (86) and a high dietary consumption of omega-3 demonstrates a lower incidence of laparoscopically confirmed endometriosis compared to individuals with a low dietary intake of omega-3 (87). A study on mice has confirmed this by exhibiting a 99-fold lower level of inflammatory cytokine IL-6 in mice with endogenously high levels of omega-3 PUFAs as well as a reduction of proliferation in endometriosis-like lesions when donor tissue was transferred to a PUFA rich host environment (88). They demonstrated that omega-3 PUFA levels influence immune, angiogenic, and proliferative factors implicated in the early establishment of endometriosis.

### Future directions

The majority of research in microbiota and endometriosis has been focused on bacteria. The most common analytic method used in these studies has been the 16sRNA sequencing. 16sRNA sequencing uses a single gene in bacteria and is used to differentiate bacterial taxa and their relative abundance. However, it does not distinguish between the different strains of each genus of bacteria. Different strains of the genus of bacteria are genomically distinct and one strain can cause significant illness whilst another strain could be considered a probiotic (7). Shotgun metagenomics and metabolomics are newer analytical methods that have become more accessible in the recent years. They examine a wider range of microbiota and microbiome, although both come with analytical limitations due to the ongoing developments in the field. Shotgun metagenomics fragments all the DNA from a sample and sequences these fragments. It can infer a complete list of microbial strains including viruses and fungi. Metabolomics is the study of the nonprotein small molecules including products of metabolism (7, 89). Further research with these methods may yield different results, especially on different types of microbiota, such as fungi or viruses and new associations with endometriosis may be discovered.

## Discussion

Microbiome testing has potential be used a non-invasive test to detect endometriosis. There is a significant delay in diagnosis of endometriosis, with the average time between onset of symptoms to diagnosis being 8 years and the range reported as 4-12 years in different studies (90–92). Previously, laparoscopic diagnosis was considered the gold standard but has the disadvantage of being invasive. As imaging techniques have improved over the years, transvaginal ultrasound and MRI for diagnosis of deep endometriosis have proven to have great accuracy and now accepted as first line diagnostic tools (1, 93). However, they are limited by the availability of skilled sonographers, sonologists and radiologists. In addition, the techniques to improve diagnosis of superficial endometriosis on ultrasound are still relatively new (94, 95). If available in the future, a simple microbiota test could complement the imaging modalities well in non-invasive diagnosis of endometriosis. Currently there is no evidence for microbiome signatures of different stages of endometriosis or predicting infertility. Further research is needed to be able to make this a possibility. Discovering the microbial signature of endometriosis would also create avenues for future research into developing methods to alter the microbiome *via* probiotics, microbial transplants, or immunomodulation to alter the disease.

## Author contributions

CU wrote the manuscript. JM, FE-A and GC outlined the content of the review, reviewed, edited and approved the final draft. GC supervised the project. All authors contributed to the article and approved the submitted version.
